# Supplementary material for: Rapid custom prototyping of soft poroelastic biosensor for simultaneous epicardial recording and imaging
Source: Nat Commun. 2021 Jun 17;12:3710. doi: 10.1038/s41467-021-23959-3 (PMC8211747; doi:10.1038/s41467-021-23959-3)
Supplement: Supplementary file 1 — Supplementary Information [file 41467_2021_23959_MOESM1_ESM.docx]

**Supplementary Information: Rapid Custom Prototyping of Soft Poroelastic Biosensor for Simultaneous Epicardial Recording and Imaging**

**Bongjoong Kim^1†^, Arvin H. Soepriatna^2†^, Woohyun Park^1^, Haesoo Moon^2^, Abigail Cox^3^, Jianchao Zhao^4^, Nevin S. Gupta^4^, Chi Hoon Park^4,5^, Kyunghun Kim^2^, Yale Jeon^2,6^, Hanmin Jang^2,6^, Dong Rip Kim^6^, Hyowon Lee^2^, Kwan-Soo Lee^4^*, Craig J. Goergen^2^*, Chi Hwan Lee^1,2,7^***

^1^School of Mechanical Engineering, Purdue University, West Lafayette, IN 47907, USA. ^2^Weldon School of Biomedical Engineering, Purdue University, West Lafayette, IN 47907, USA. ^3^Department of Comparative Pathobiology, Purdue College of Veterinary Medicine, West Lafayette, IN, USA. ^4^Chemical Diagnostics and Engineering, Los Alamos National Laboratory, Los Alamos, New Mexico 87545, USA. ^5^Department of Energy Engineering, Gyeongnam National University of Science and Technology, Jinju-Si 660-758, Republic of Korea. ^6^School of Mechanical Engineering, Hanyang University, Seoul 04763, Republic of Korea. ^7^Department of Materials Engineering, Purdue University, West Lafayette, IN 47907, USA. ^†^These authors contributed equally to this work. *Correspondence and requests for materials should be addressed to C.H.L. (email: [lee2270@purdue.edu](mailto:lee2270@purdue.edu)) or C.J.G. (email: [cgoergen@purdue.edu](mailto:cgoergen@purdue.edu)) or K.-S.L. (email: kslee@lanl.gov).

**
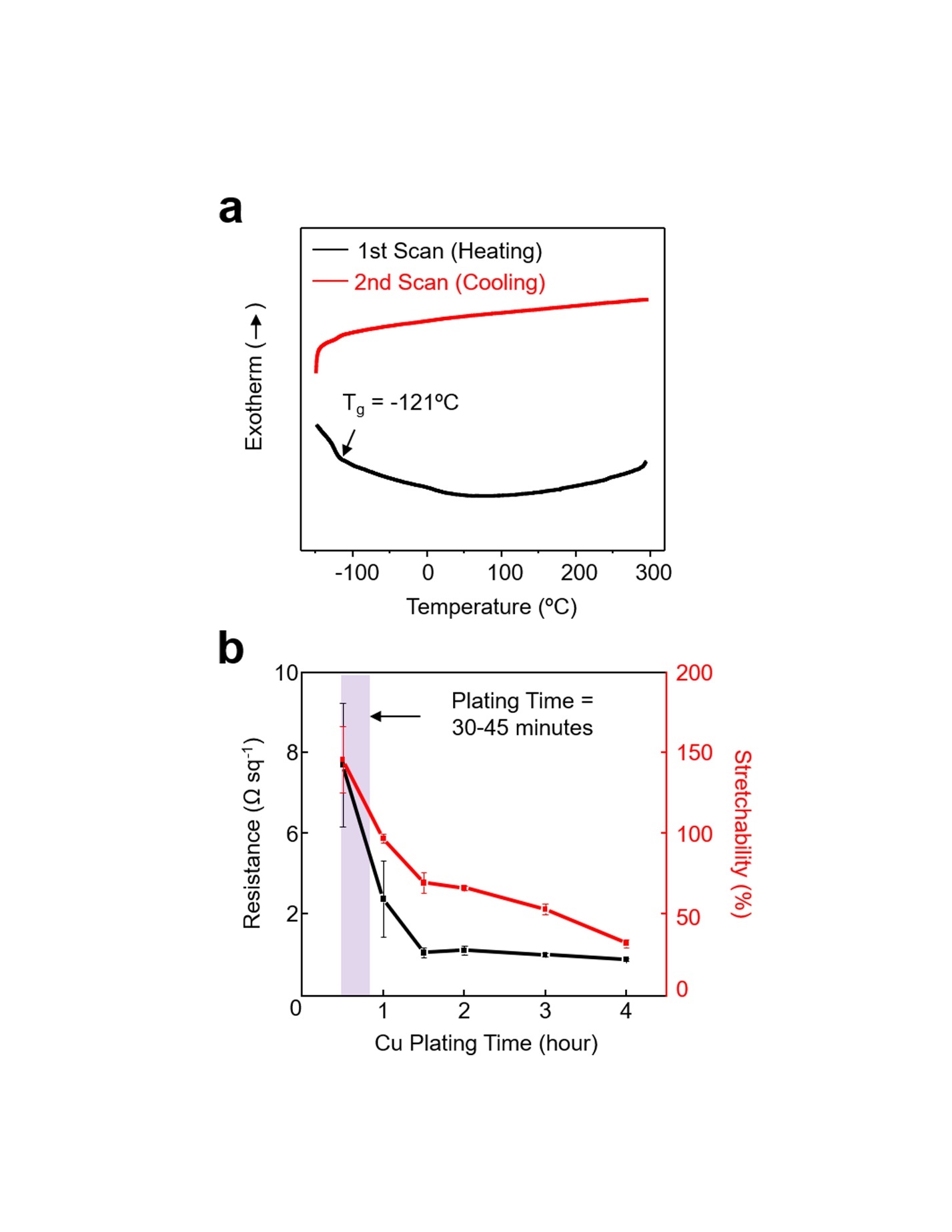
**

**Supplementary Figure 1.** (a) Results of differential scanning calorimetry (DSC) for the sponge-like foam. (b) Change in the resistance and stretchability of the custom-printed sensor array with respect to the plating time of Cu (n = 5 per time). The error bars represent the standard deviation.

**
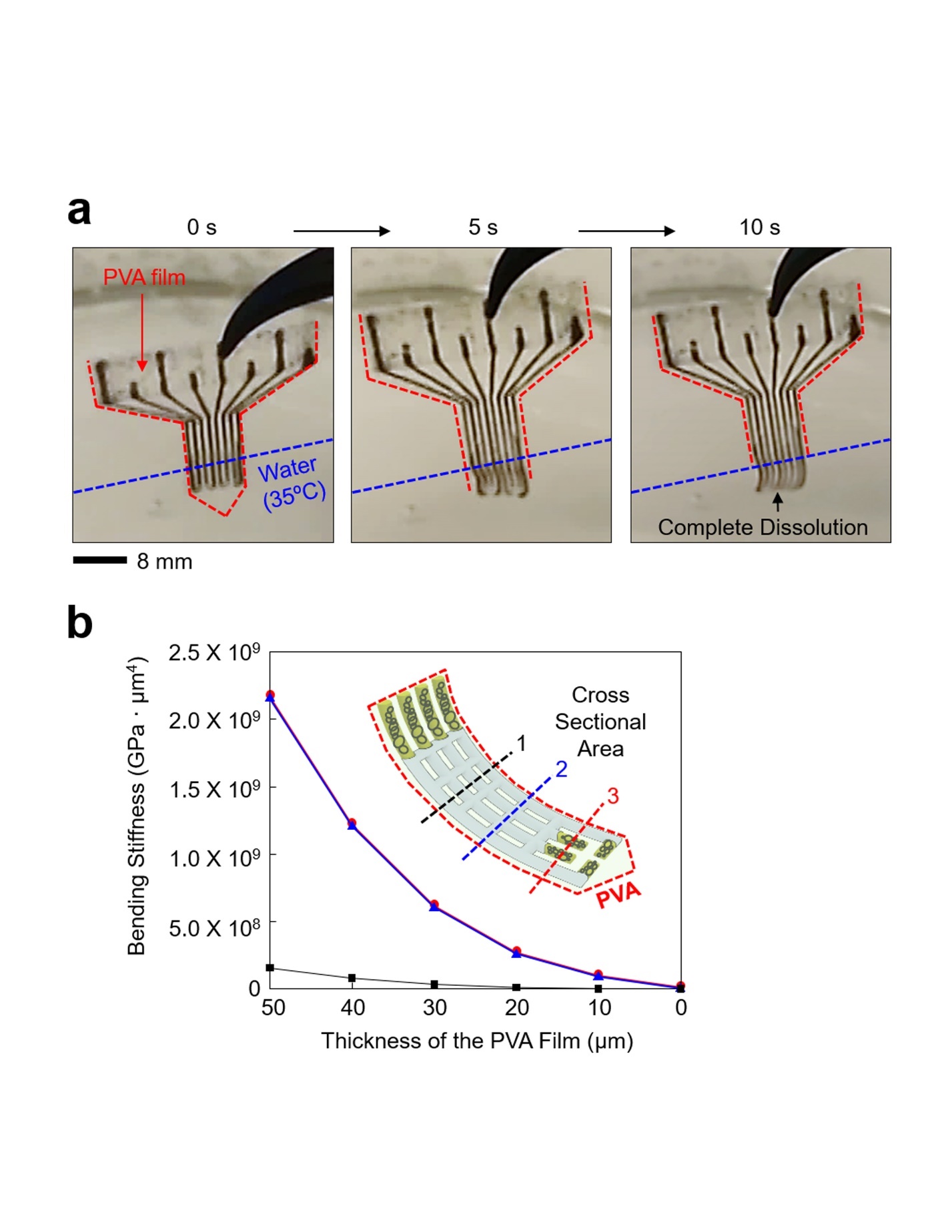
**

**Supplementary Figure 2.** (a) Photographs of the custom-printed sensor array immersed in a warm water bath (35ºC) for 10 seconds. (b) Results of the bending stiffness of the custom-printed sensor array with respect to the thickness of the water-soluble film.

**
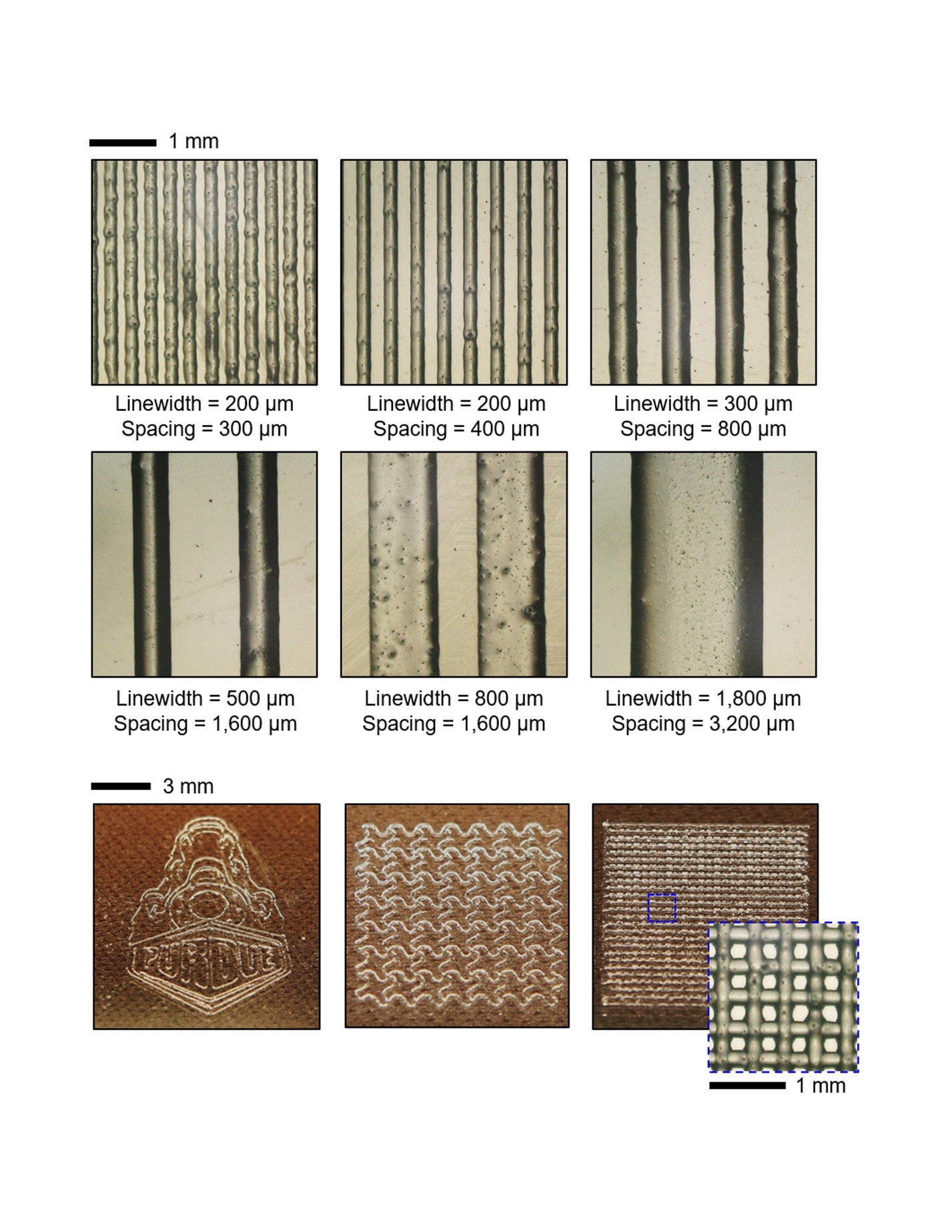
**

**Supplementary Figure 3.** Photographs of various as-printed structures using the formulated ink with the weight ratio of 6.0:3.3:0.7.

**
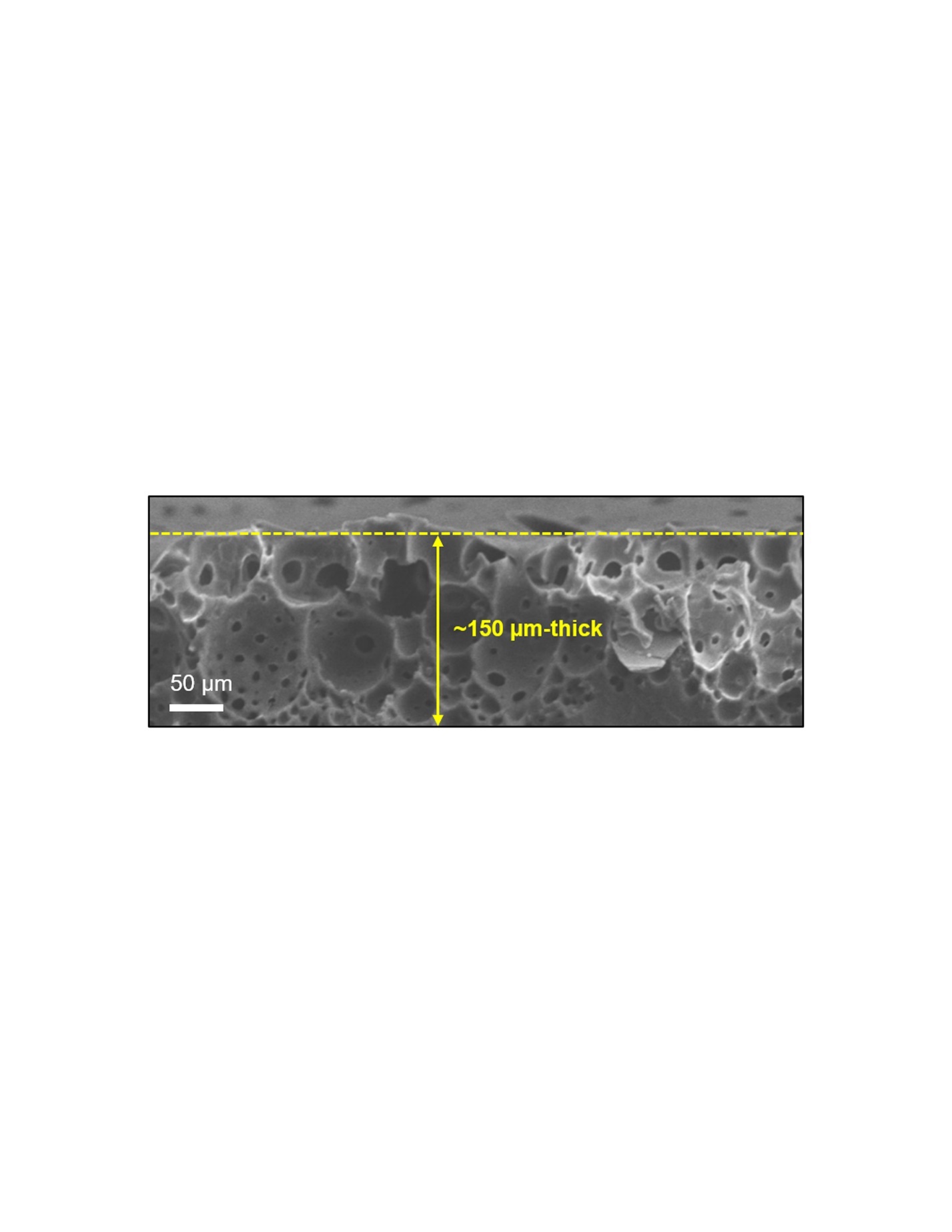
**

**Supplementary Figure 4.** Cross-sectional side view of a SEM image for the sponge-like foam.

**
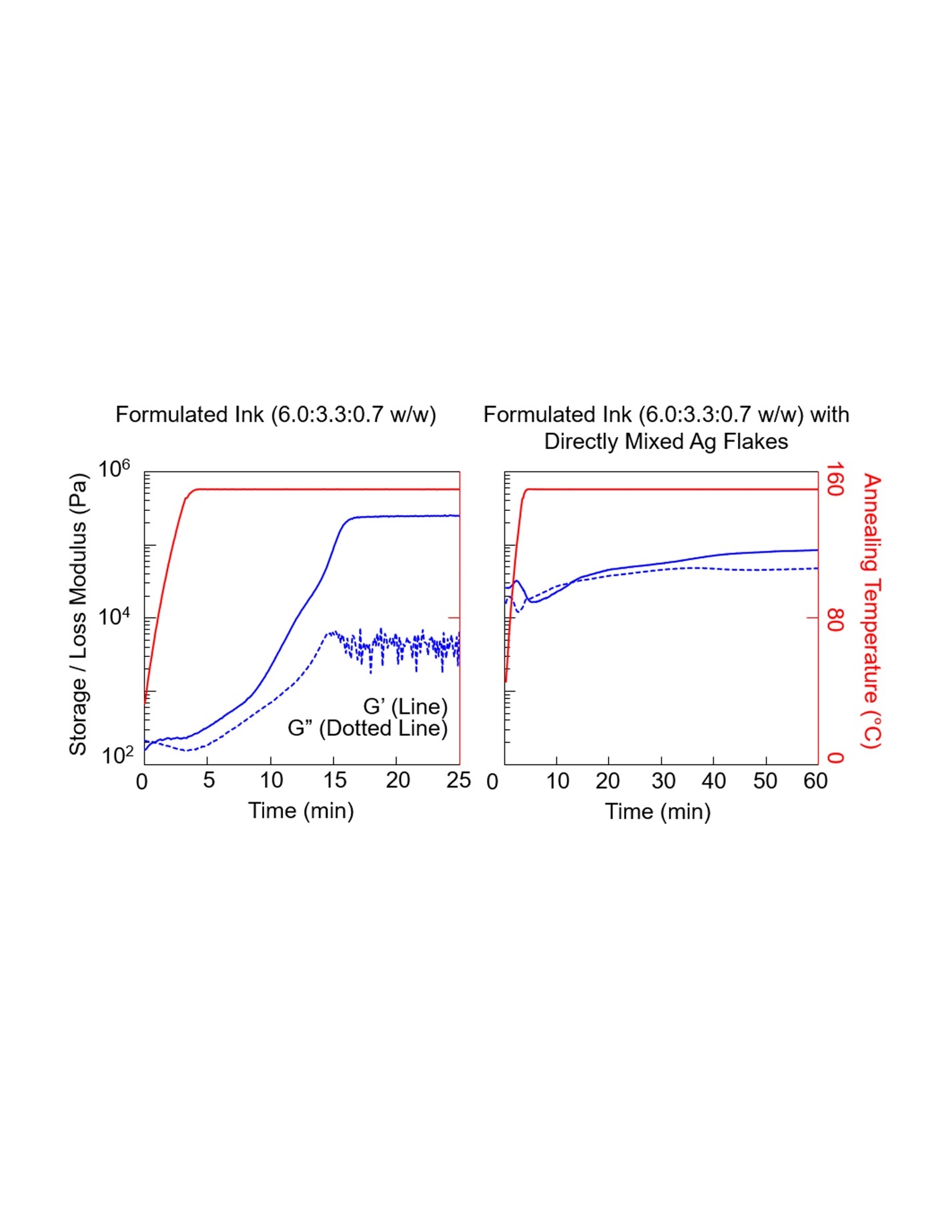
**

**Supplementary Figure 5.** Change in the storage and loss modulus of the formulated ink with the weight ratio of 6.0:3.3:0.7 (left panel) and the ink with directly mixed Ag flakes (right panel) over time.

**
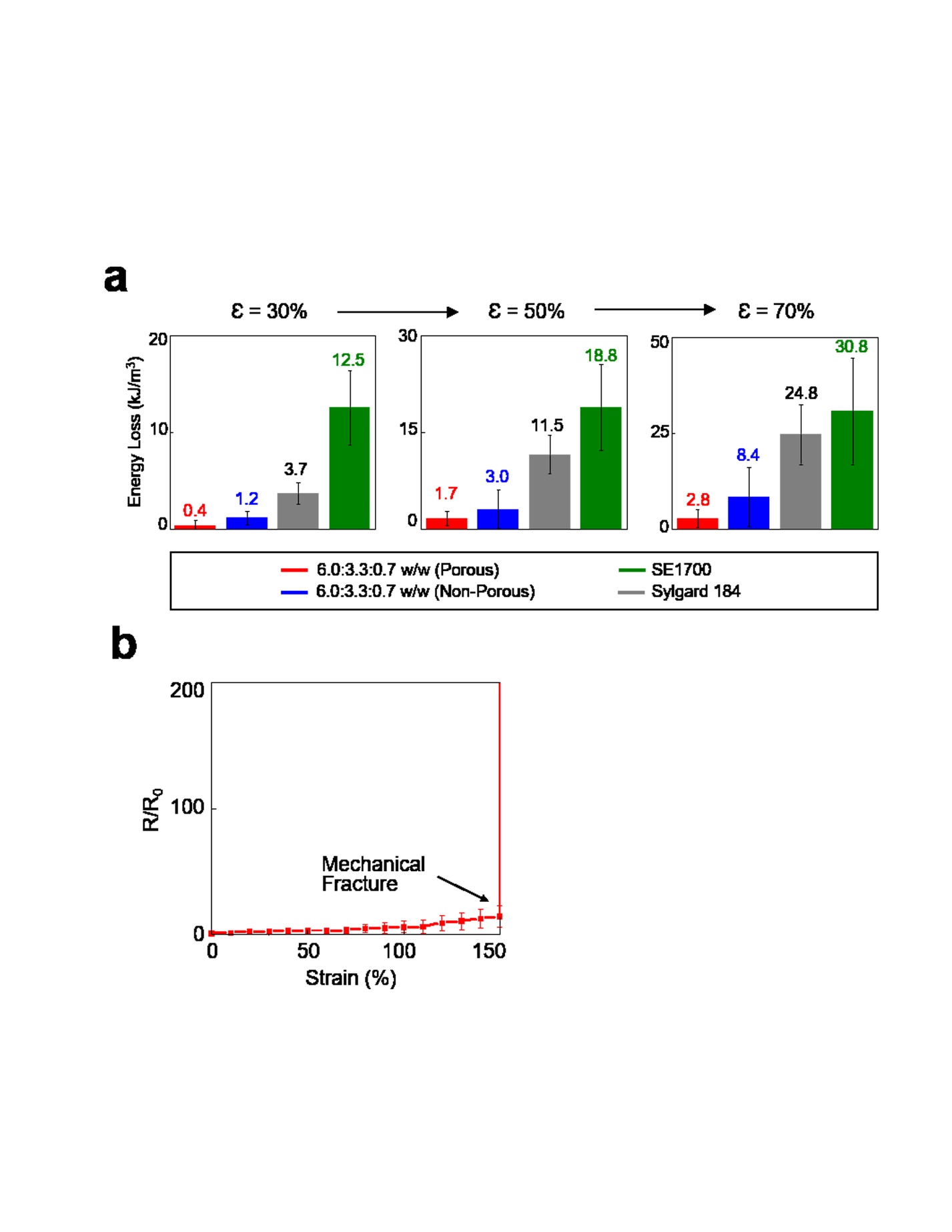
**

**Supplementary Figure 6.** (a) Change in the energy loss of the custom-printed sensor array with respect to a strain ranging from 30% to 70%. (n = 5 per group) (b) Change in the resistance of the custom-printed sensor array with respect to the strain up to 150% (n = 4 per group). The error bars represent the standard deviation.


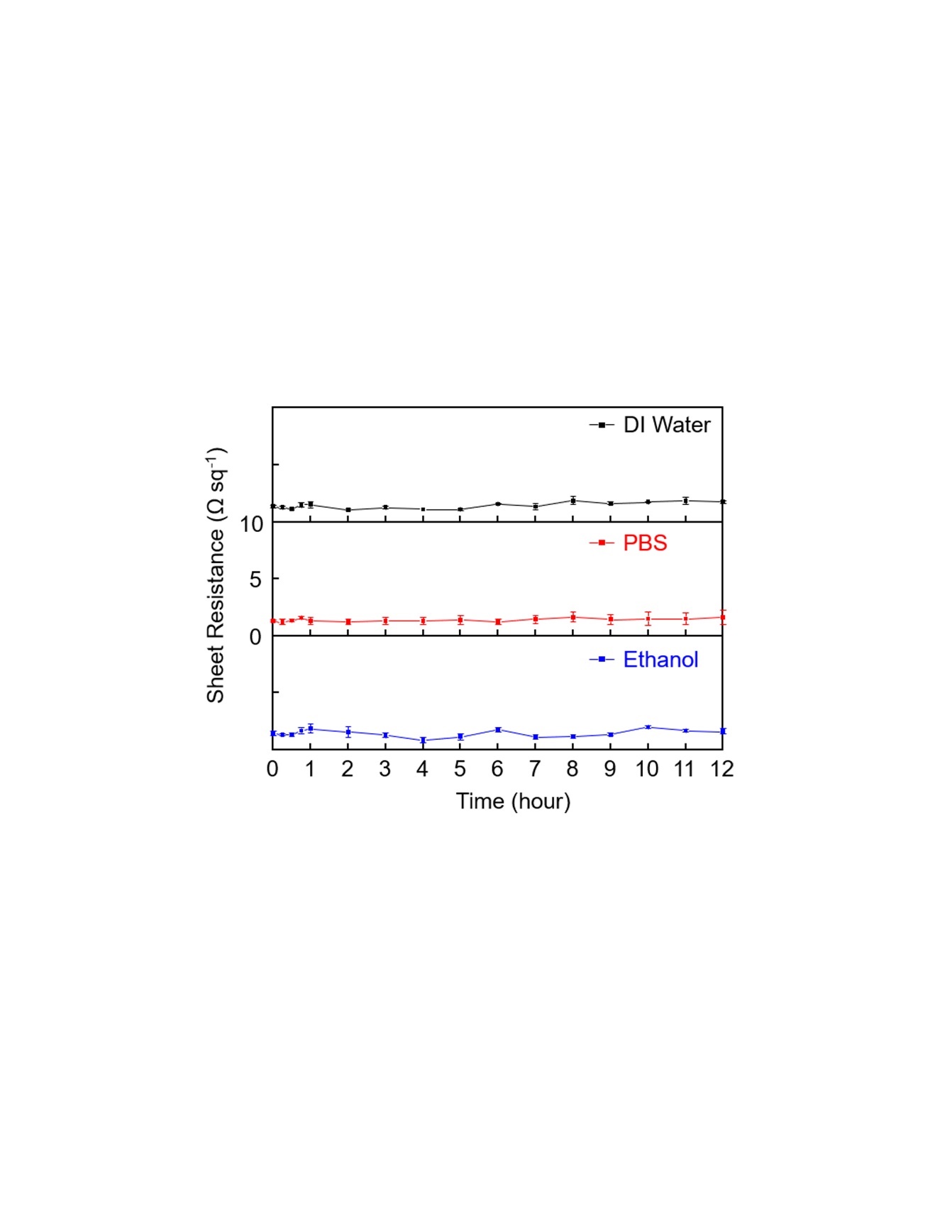


**Supplementary Figure 7.** Change in the sheet resistance of the devices soaked in a bath of DI water (top panel), PBS (middle panel), and ethanol (bottom panel) for 12 hours (n = 3 per group). The error bars represent the standard deviation.


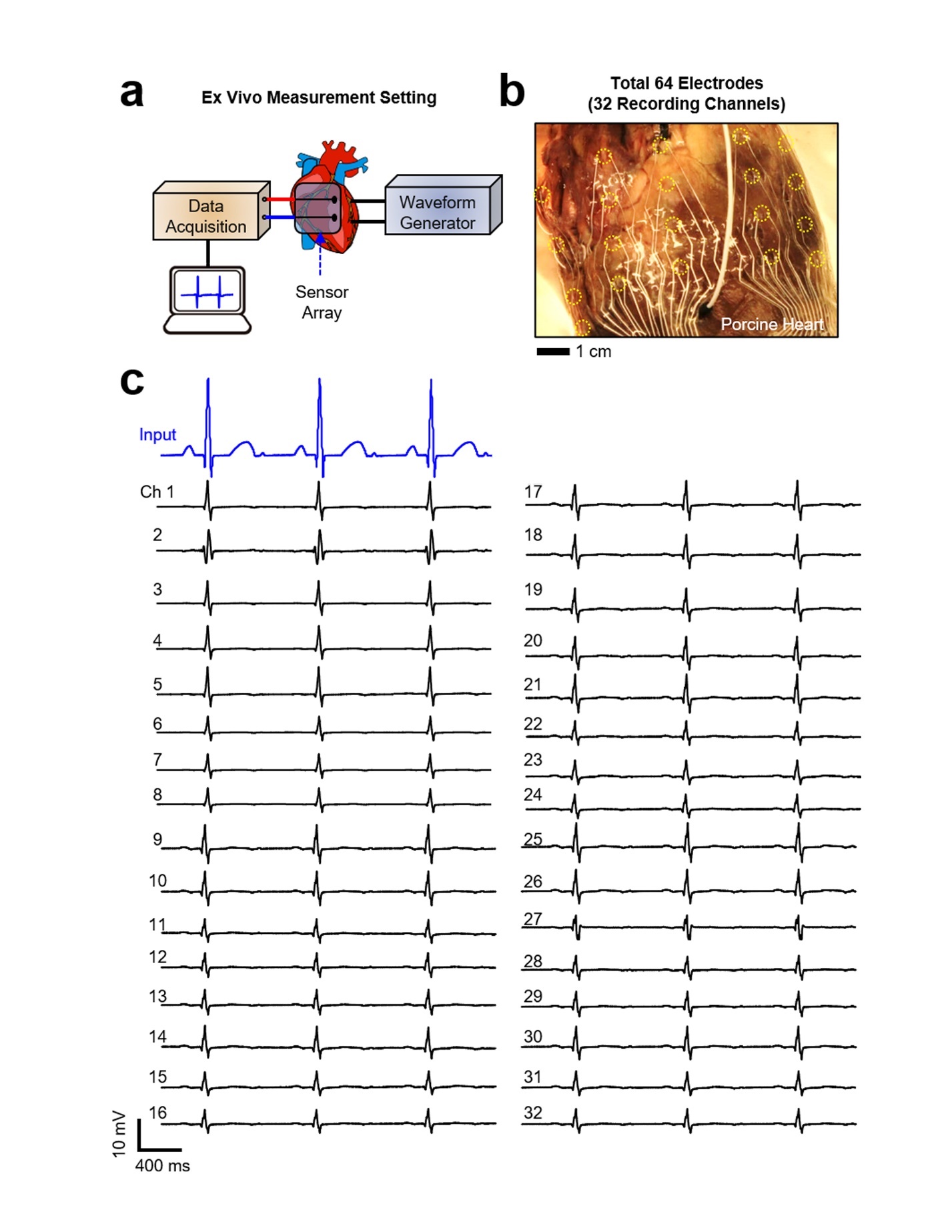


**Supplementary Figure 8.** (a) Schematic illustration of an experiment setup for the ex vivo measurement of epicardial ECG signals from the enucleated porcine heart. (b) Photograph of the device (total 64 electrodes) placed on the surface of the enucleated porcine heart. (c) Measurement results of the epicardial ECG signals by applying an artificial ECG waveform.


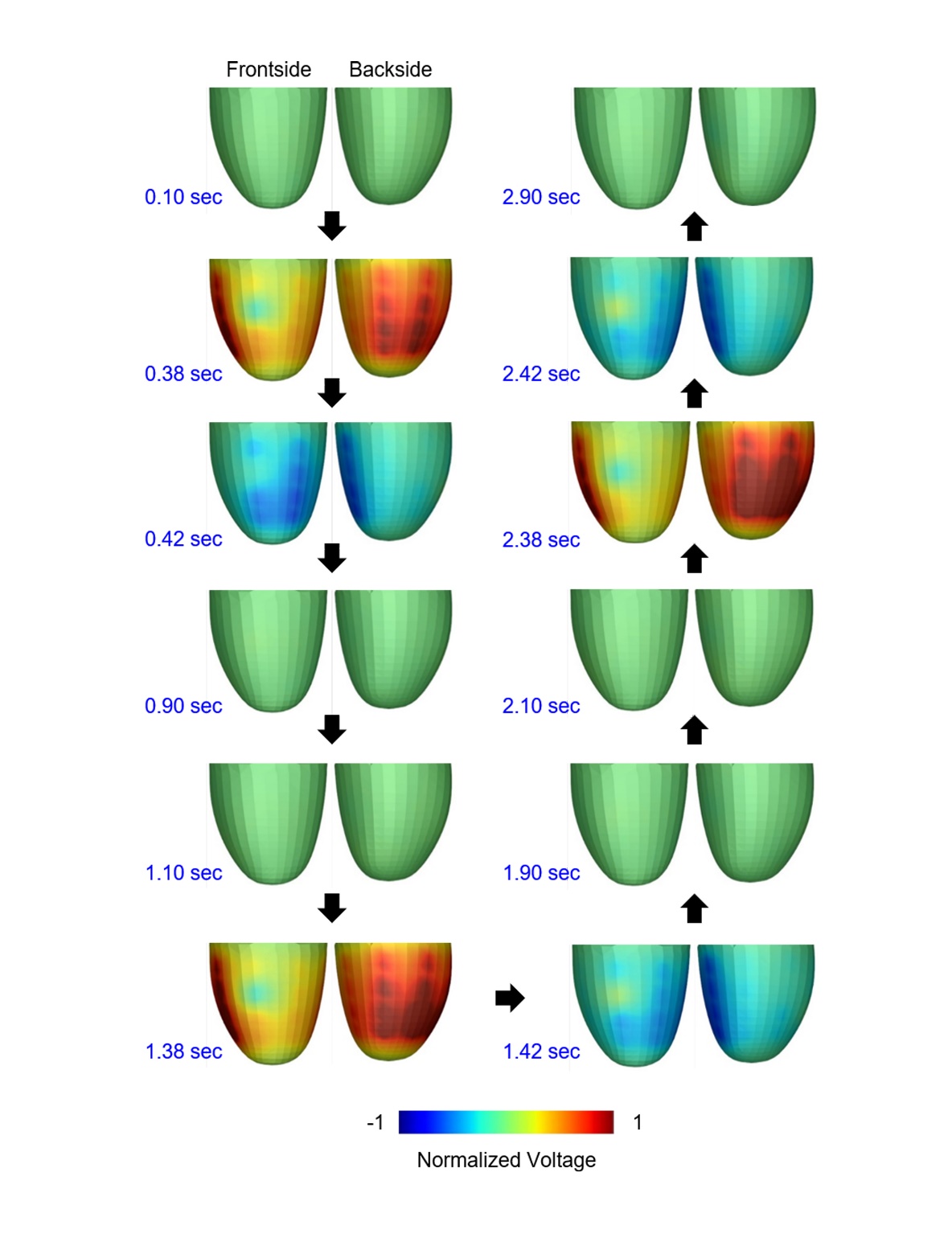


**Supplementary Figure 9.** Spatiotemporal ECG mapping results for the ex vivo measurement of epicardial ECG signals from the enucleated porcine heart.

**
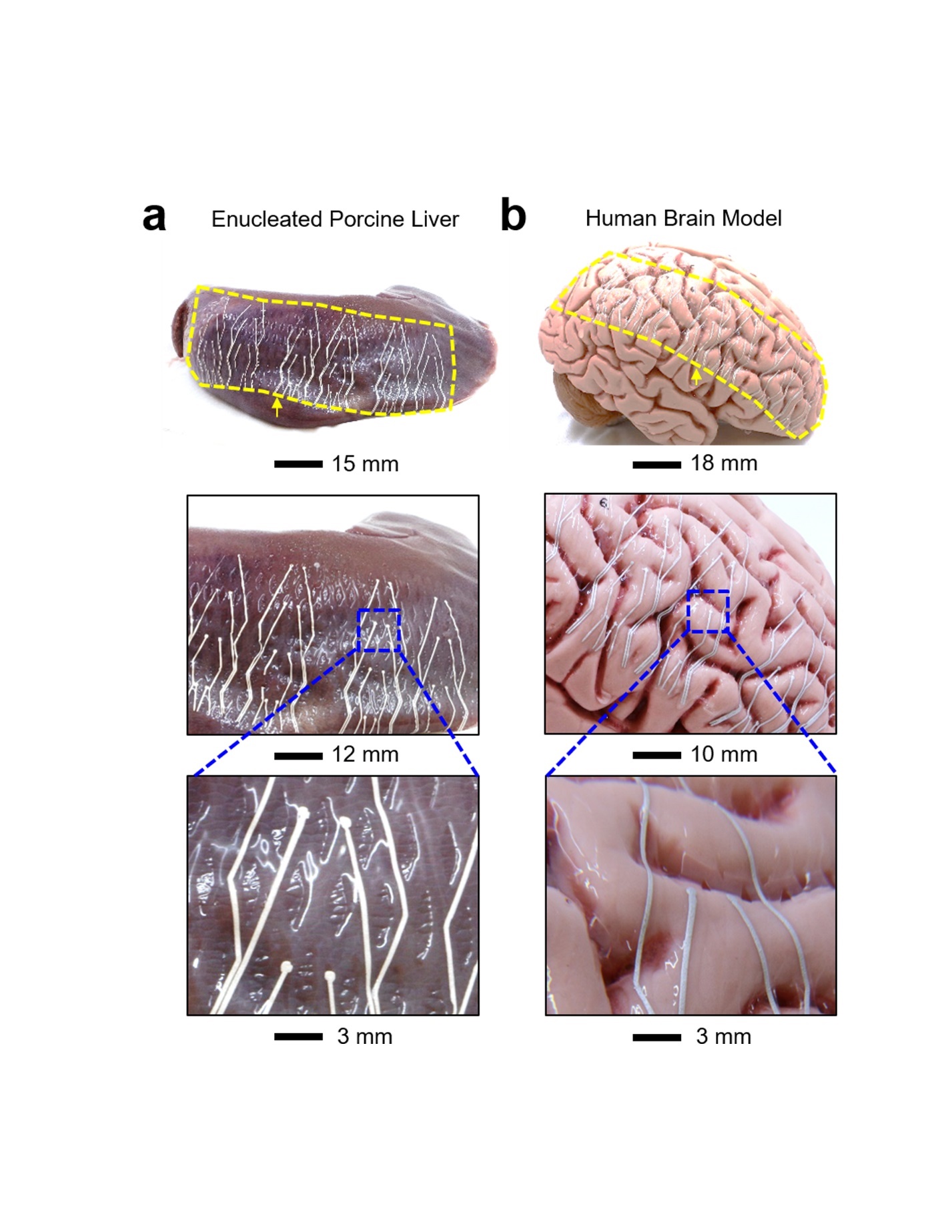
**

**Supplementary Figure 10.** Photographs of the devices placed on the surface of (a) the enucleated porcine liver and (b) a human brain model.

**
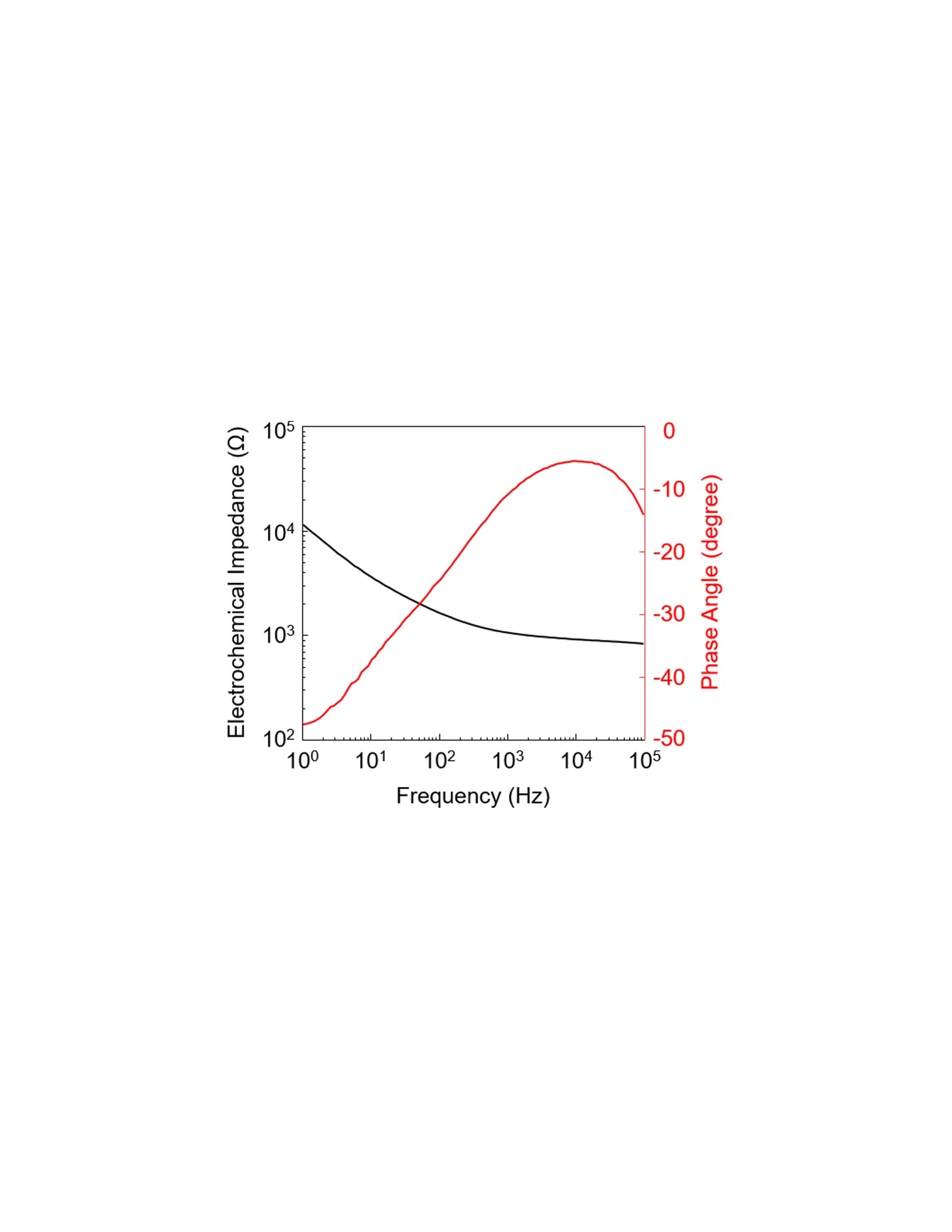
**

**Supplementary Figure 11.** Electrochemical impedance of the device soaked in a bath of PBS as a function of frequency.

**
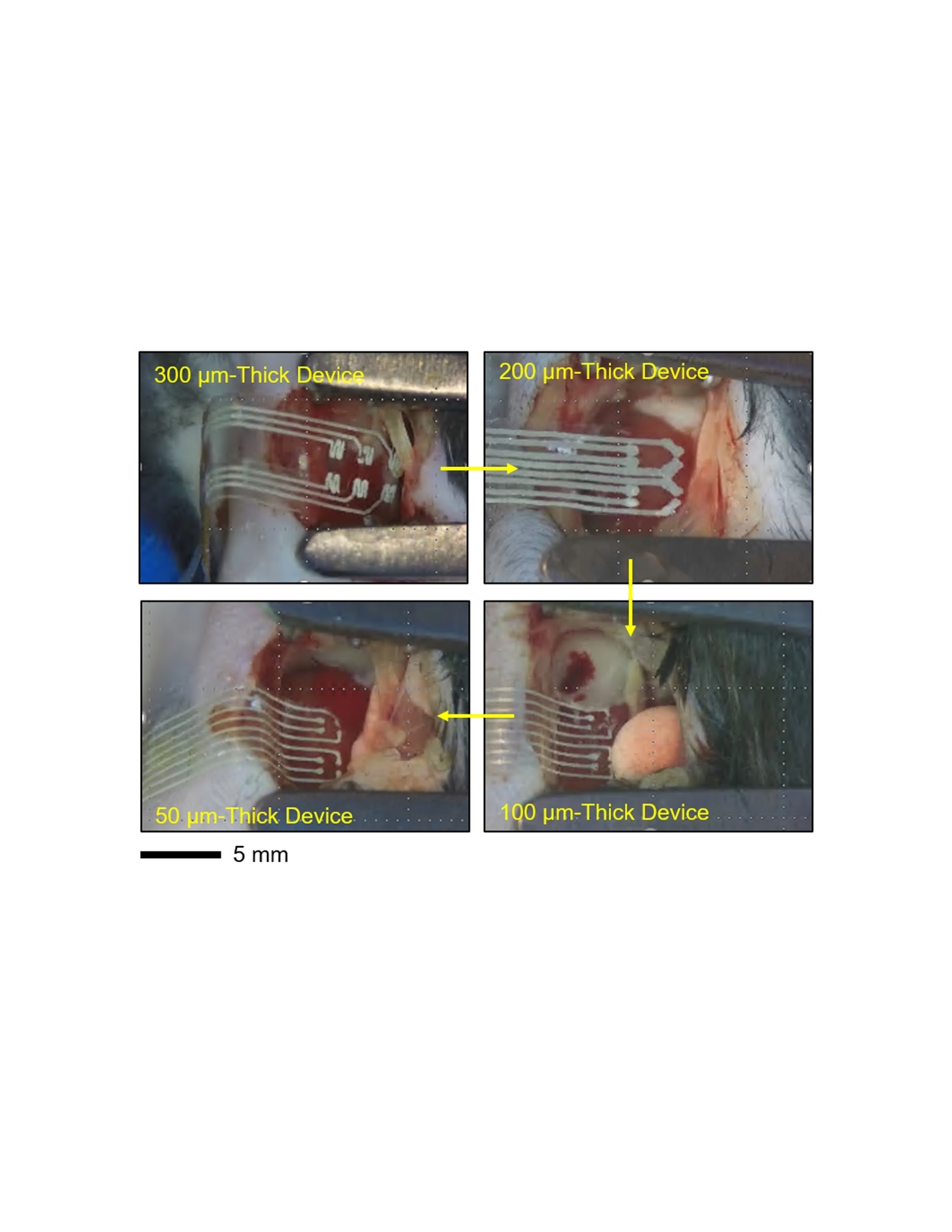
**

**Supplementary Figure 12.** Photographs of the devices with different thicknesses ranging from 50 µm to 300 µm placed on the epicardial surface of a murine heart.


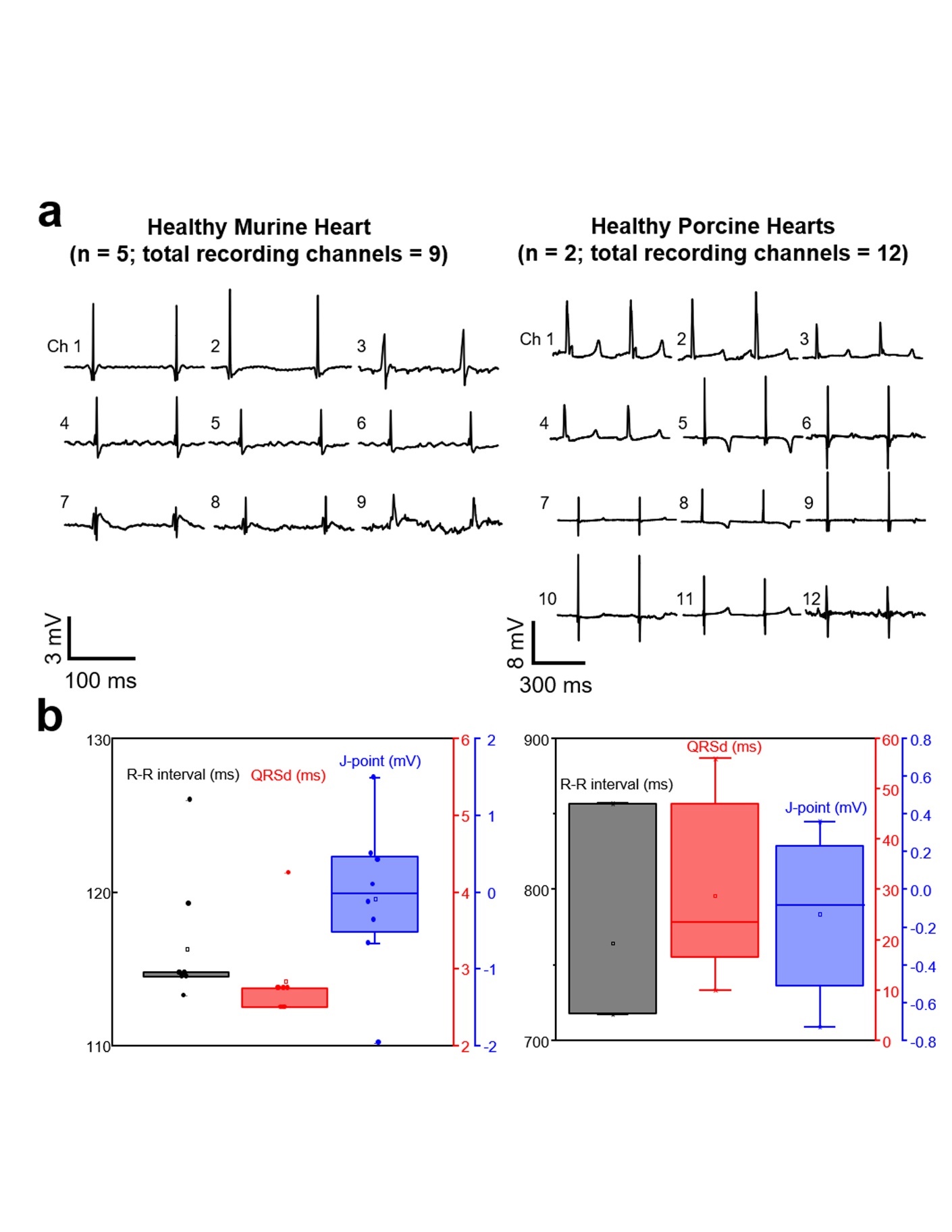


**Supplementary Figure 13.** (a) Epicardial ECG signals obtained from the healthy murine (n = 5) and porcine hearts (n = 2). (b) The corresponding quantitative data of R-R interval, QRS duration, and J-point elevation of the 9 channels and 12 channels from the healthy murine and porcine hearts, respectively. Box plots indicate median (middle line), 25th, 75th percentile (box) and whiskers represent the outliers (coefficient 1.5) of the distribution. The mean (square), maximum and minimum values of the distribution are also shown.

**
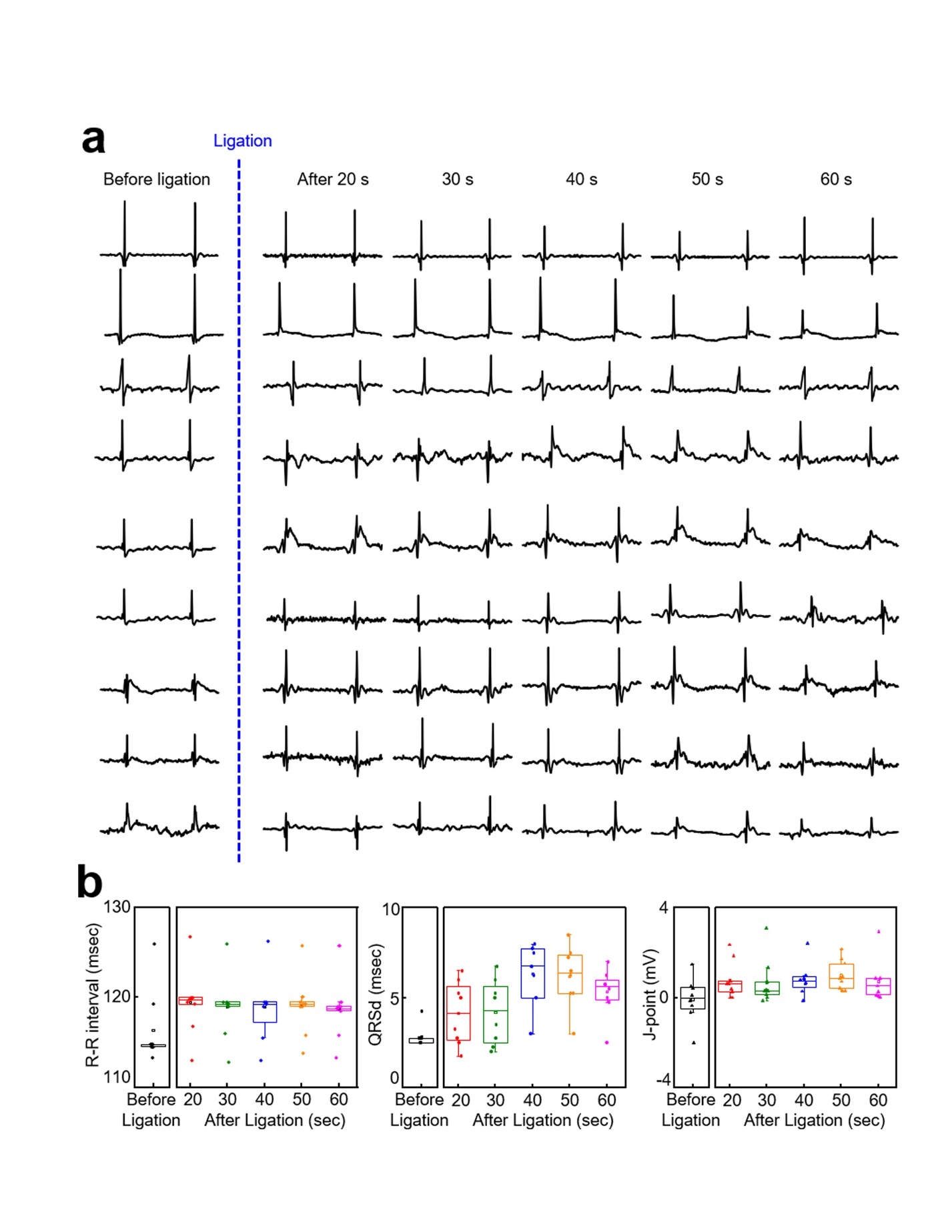
**

**Supplementary Figure 14.** (a) Epicardial ECG signals obtained from a murine myocardial infarction model (n = 5). (b) The corresponding quantitative data of R-R interval (left panel), QRS duration (middle panel), and J-point elevation (right panel) (n = 9). Box plots indicate median (middle line), 25th, 75th percentile (box) and whiskers represent the outliers (coefficient 1.5) of the distribution. The mean (square), maximum and minimum values of the distribution are also shown.

**
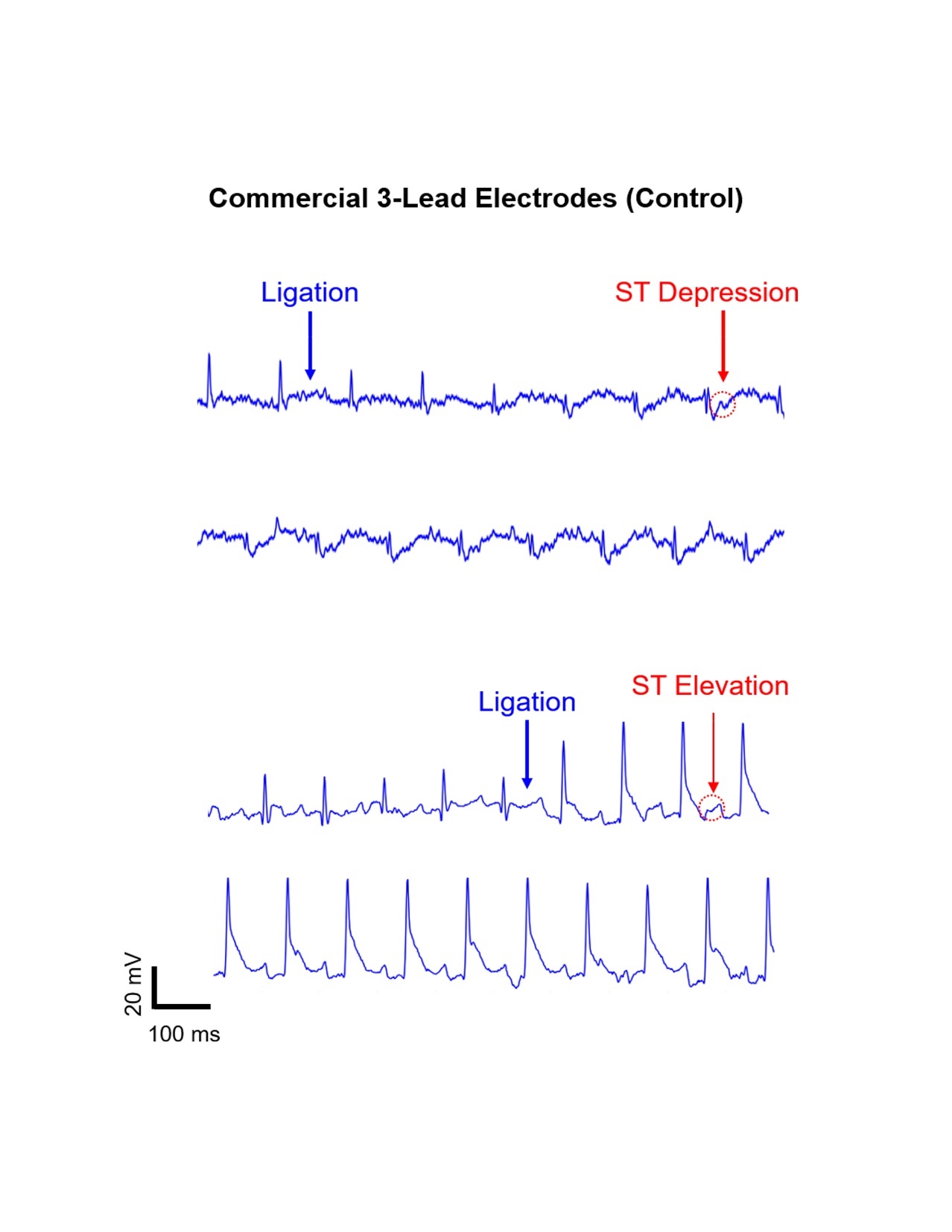
**

**Supplementary Figure 15.** Representative global ECG data of ST-segment depression (top panel) and elevation (bottom panel) within seconds following left coronary artery ligation (blue arrows).

**
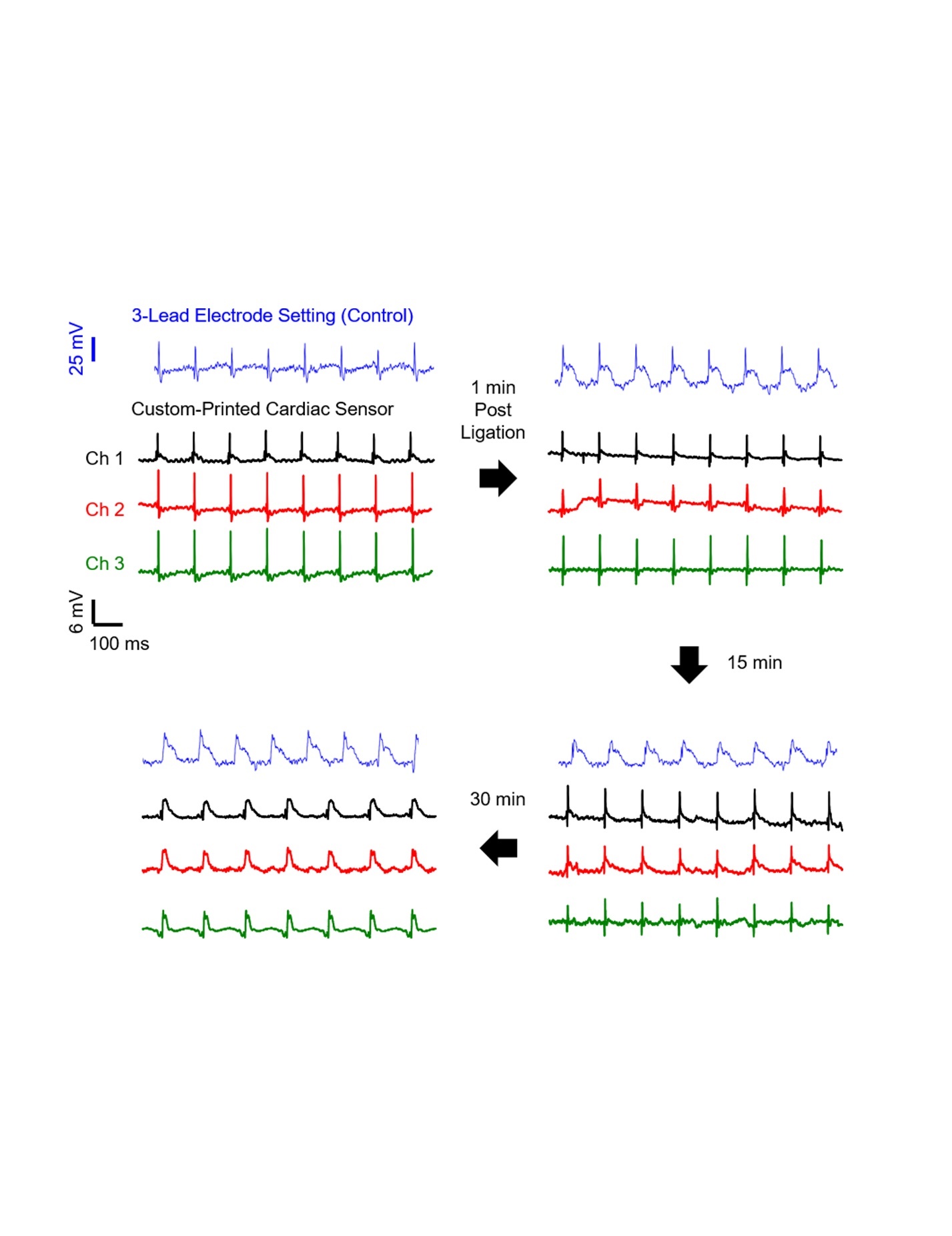
**

**Supplementary Figure 16.** Representative ECG data with persistent ST-segment elevation at baseline and at 1, 15, and 30 minutes following left coronary artery ligation.

**
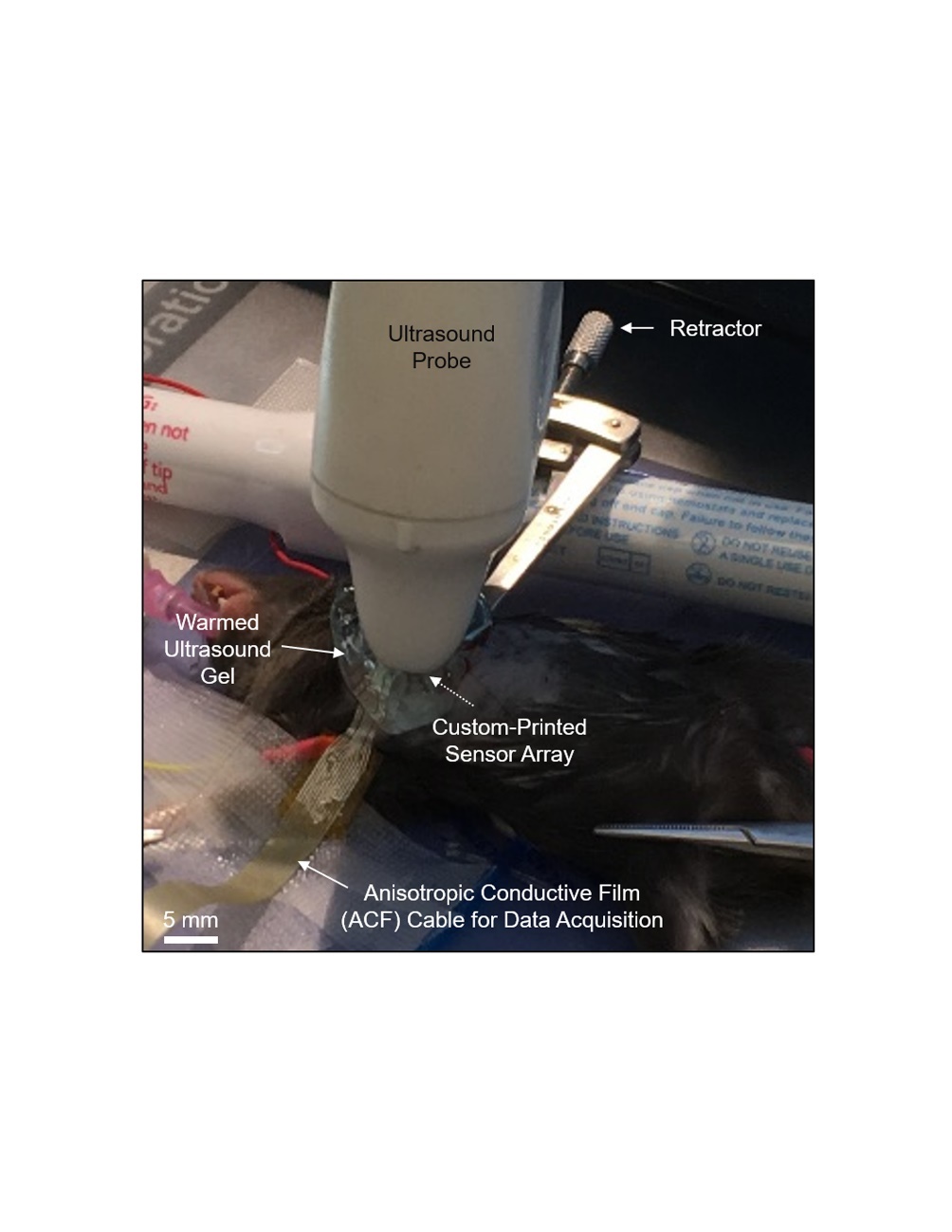
**

**Supplementary Figure 17.** Photograph of the measurement setup for simultaneous epicardial ECG recording and ultrasound imaging.

**
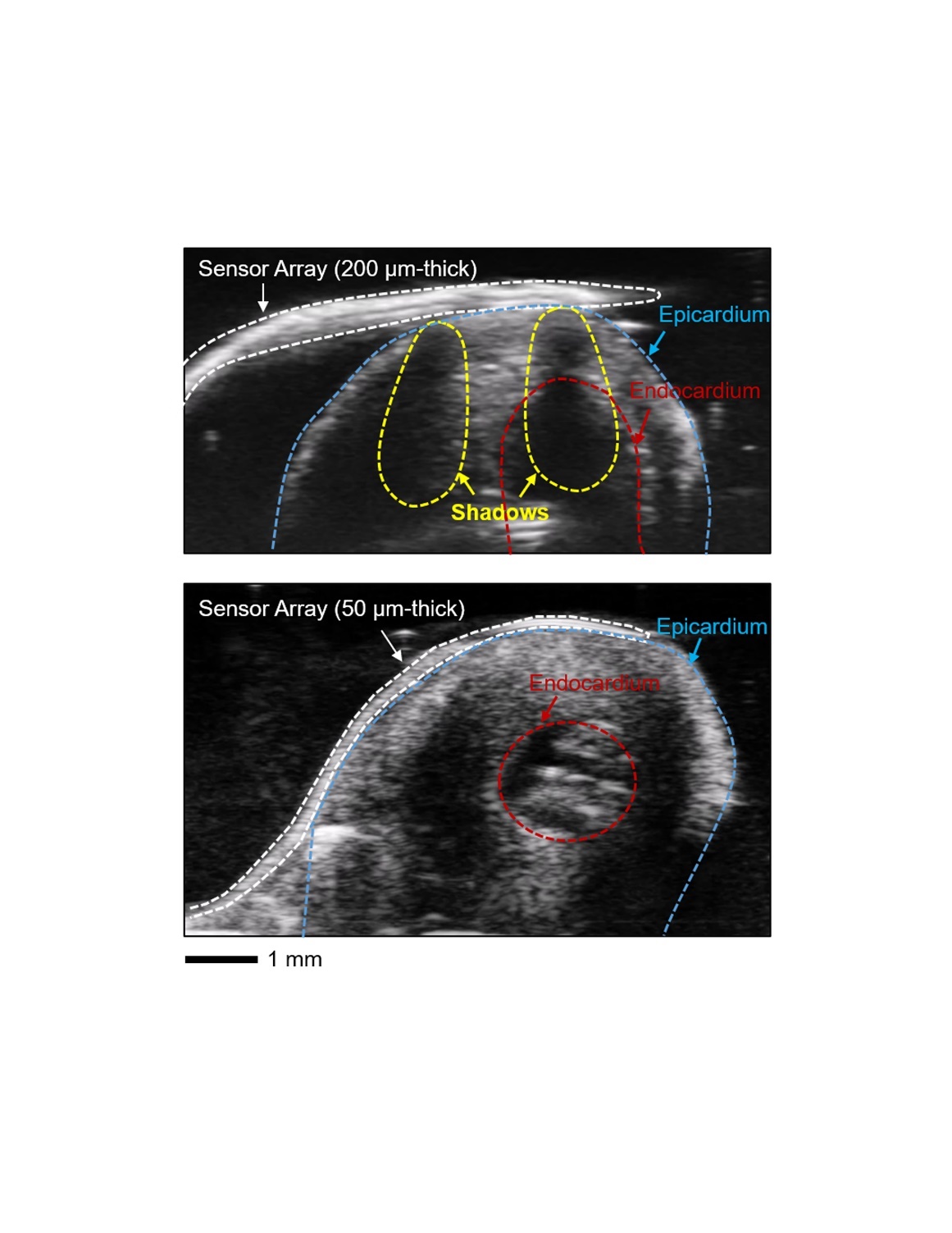
**

**Supplementary Figure 18.** Representative ultrasound images of relatively thick (200 µm-thick; top panel) and thin (50 µm-thick; bottom panel) devices placed on the epicardial surface of a fixed murine heart.

**
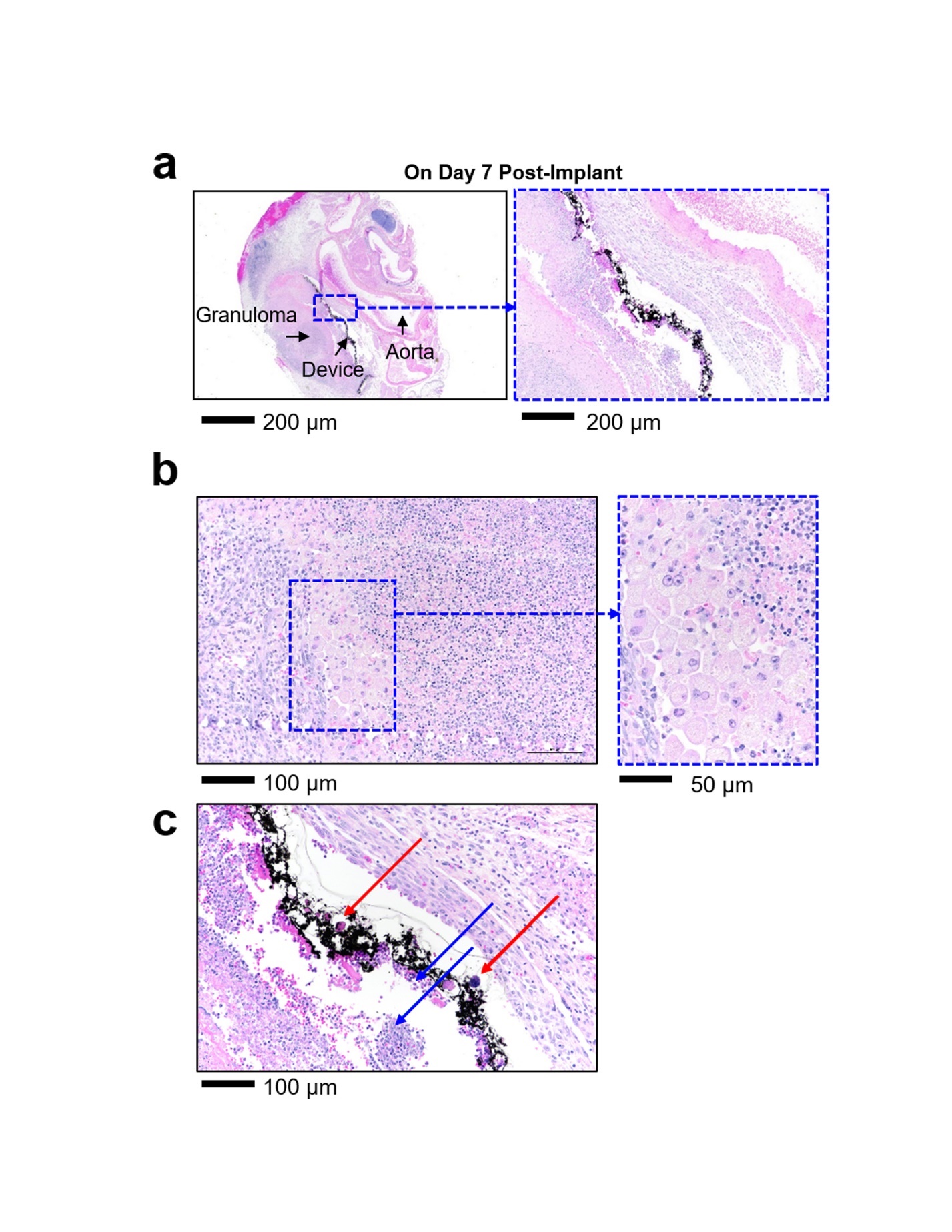
**

**Supplementary Figure 19.** (a) Overview of granuloma and aorta on day 7 post-implant. (b) Magnified views (at 20×) of granuloma formation on day 7 post-implant. The boxed area identifies the presence of macrophages, multi-nucleated giant cells, and fibroblasts. The inset image demonstrates macrophages containing phagocytized debris resulting from the intralesional device. (c) Magnified views (20×) of macrophages and multi-nucleated giant cells at the surface of the implanted device (red arrows). The neutrophils (blue arrows) suggest acute to chronic inflammation directed at the device.

**
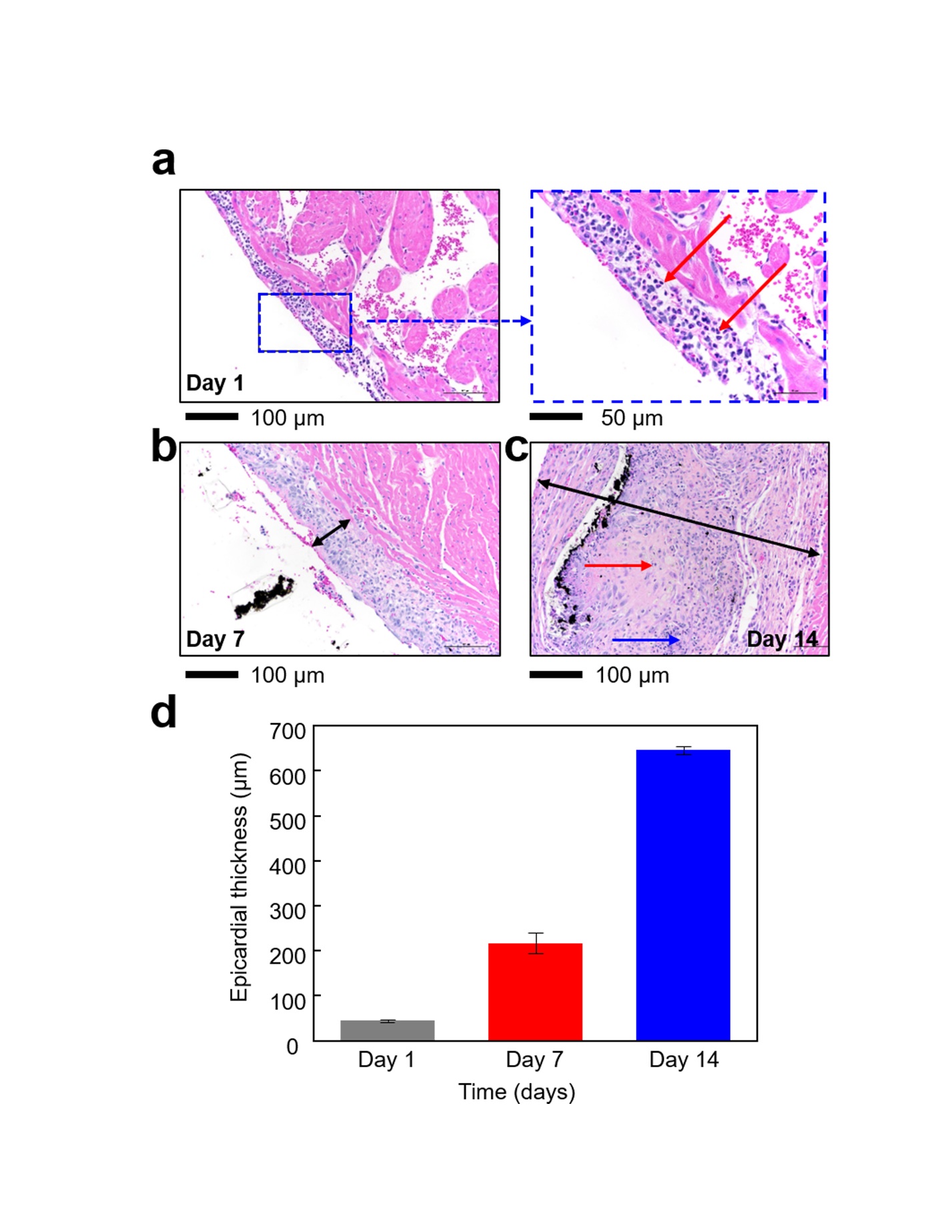
**

**Supplementary Figure 20.** (a) Day 1 epicardium at 20 × (left) and 40 × (right) magnification. The epicardium presents at five-times normal thickness and is comprised of neutrophils (red arrows). (b) Day 7 epicardium. The double-headed line indicates a thickening of epicardium by mononuclear cells and fibroblasts due to chronic inflammatory response. (c) Day 14 epicardium. The double-headed line indicates a worsening of epicardial thickening. The presence and epithelioid morphology of macrophages (red arrow) indicate chronic granulomatous response (e.g., foreign body response) at the surface of the implanted device. The neutrophils (blue arrow) suggests that acute chronic inflammation directed towards the implanted device. (d) Progression of epicardial thickness measured using ImageJ (n = 5). The measurements were spaced at 100 µm perpendicularly from the epicardial surface to the underlying muscle layer. The error bars represent the standard deviation.


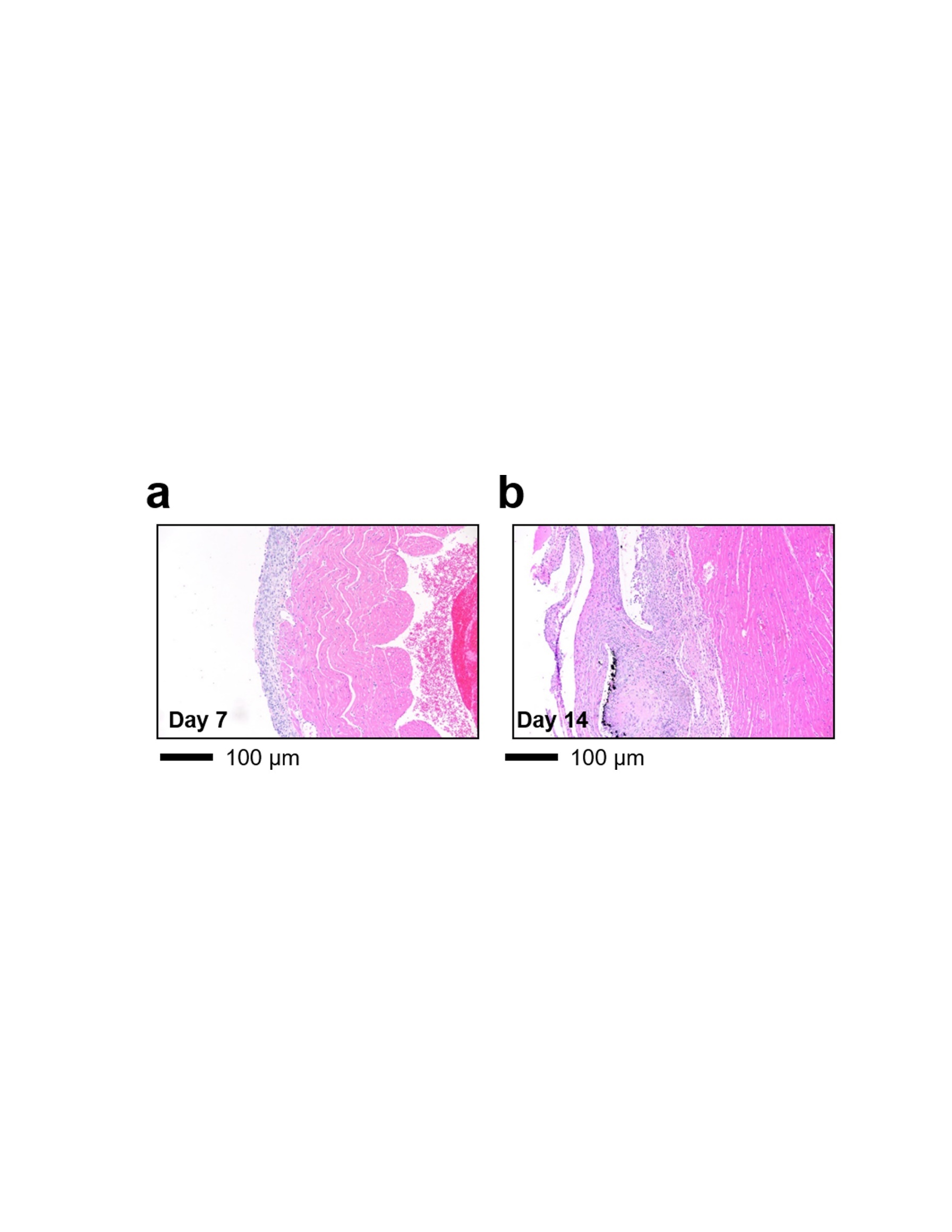


**Supplementary Figure 21.** (a) Chronic epicarditis observed on day 7 post-implant. (b) Pericardial to epicardial adhesion with intralesional device observed on day 14 post-implant.


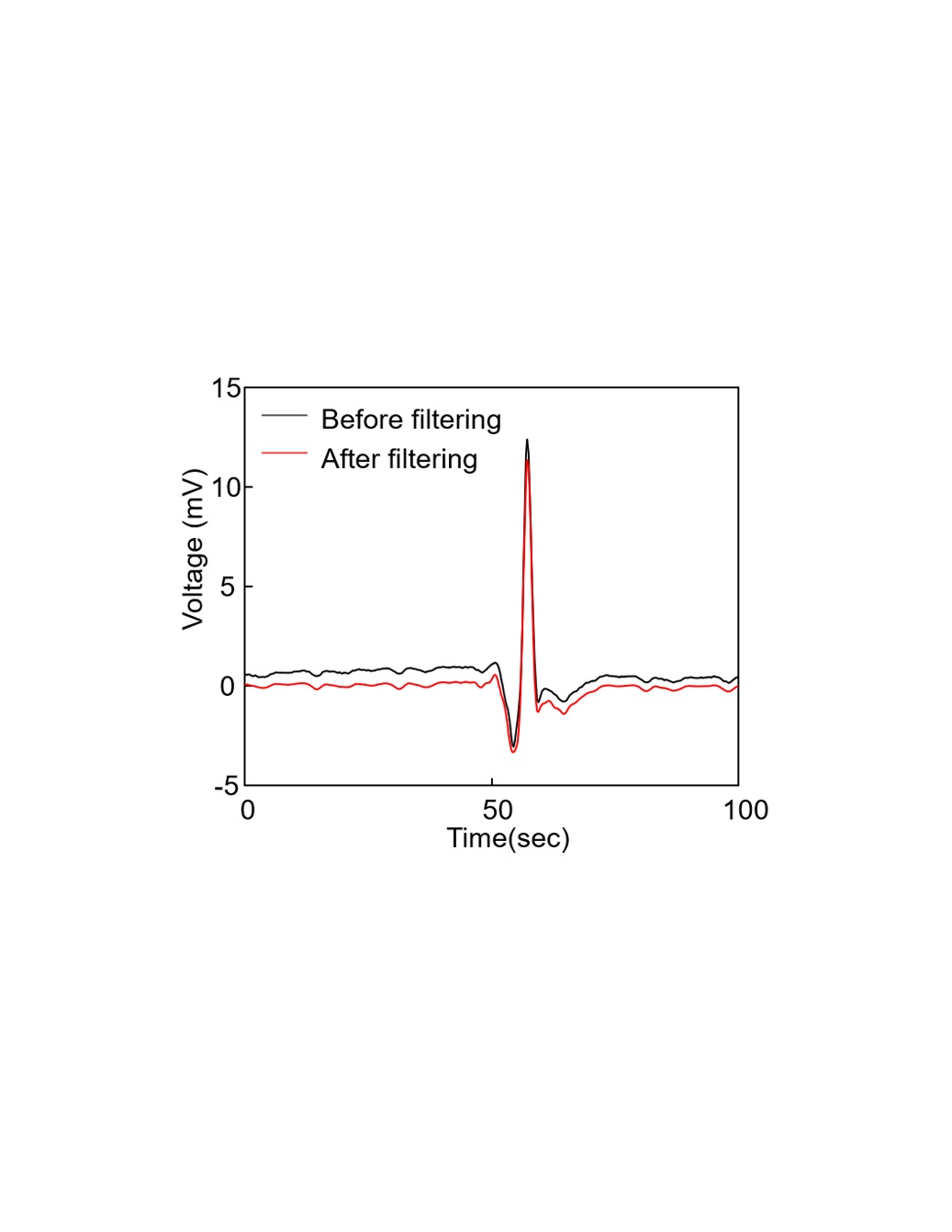


**Supplementary Figure 22.** Representative ECG data before (black line) and after (red line) high-pass filtering at 5 Hz.


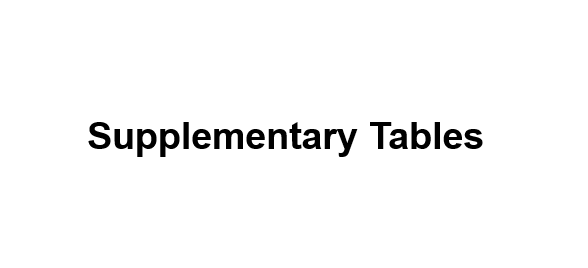


**Supplementary Table 1.** Comparisons of the poroelastic silicone composite with other existing materials in terms of the mechanical and electrical properties. The following abbreviations are used in this table. Nanowire (NW); Carbon Nanotube (CNT); Poly(Ethylene Oxide) (PEO); Carbon Black (CB); Thermoplastic PolyUrethane (TPU); Styrene-Butadiene-Styrene (SBS); Polydimethylsiloxane (PDMS)

| Patterning  Process | Materials | Conductivity | Maximum Stretchability | Young’s Modulus | Ref |
| --- | --- | --- | --- | --- | --- |
| Direct Ink Writing | Poroelastic Biosensor (This Work) | 7.72 ± 1.52 Ω∙sq^-1^ | 150% | 0.15 MPa |  |
|  | Ag Flakes/PEO | 13,800 S∙cm^−1^ | 300 % | 0.4 MPa | 2 |
|  | CB/TPU | 0.841 S∙cm^−1^ | - | 8.8 MPa | 3 |
|  | Ag/PA | 15,200 S∙cm^−1^ | - | - |  |
|  | Ag Flakes/TPU | 10^4^ S∙cm^−1^ | 240% | 2.3 MPa | 7 |
|  | Ag/Dragon Skin | 500 Ω | 250% | 0.8 MPa | 17 |
| Moulding | Ag NWs/SBS | 11,210 S∙cm^−1^ | 50% | 40 MPa | 24 |
|  | Ag-Au NWs/SBS | 72,600 S∙cm^−1^ | 840% | 37.4 MPa | 32 |
| Photolithography | Graphene-Ag NWs/PDMS | 33 Ω∙sq^−1^ | 100% | - | 9 |
|  | Ag NWs/PDMS | 26.1 Ω∙sq^−1^ | 73% | - | 10 |
|  | Au/Ni/PDMS | 1.9 Ω∙sq^−1^ | 80% | - | 25 |
| Screen Printing | Ag Flakes/Fluoroelastomer | 0.06 Ω∙sq^−1^ | 450% | - | 5 |
|  | CNTs/Fluorinated rubber | 57 S cm^−1^ | 134% | - | 21 |
| Mask Patterning | Ag NWs/PDMS | 7.5 Ω | 70% | 6.32 MPa | 11 |
|  | Au-TiO_2_ NWs/PDMS | 0.63 Ω∙sq^−1^ | 100% | - | 12 |
|  | Ag Flakes/PDMS | 5,695 S∙cm^−1^ | 80% | - | 13 |

**Supplementary Table 2.** Comparison of the MD simulation results for the interfacial interaction energy of a SiO_2_-PS particle and a SiO_2_-OH particle with the surrounding resins.

| Silica Type | Surface Property | Interaction Energy (kcal/mol) | | | Interaction Energy  Per Area  (kcal/mol Å^2^) | Interaction Energy Per Silica Weight  (kcal/mol g-silica) |
| --- | --- | --- | --- | --- | --- | --- |
|  |  | Total  Energy | Van der Waals  Energy | Electrostatic  Energy |  |  |
| SiO_2_-PS | Superhydrophobic | -119.1 | -118.8 | -0.3 | -0.1610 | -1.852 ⅹ10^21^ |
| SiO_2_-OH | Hydrophilic | -160.4 | -149.3 | -8.0 | -0.2144 | -6.969 ⅹ10^21^ |
