# Supplementary material for: Rapid custom prototyping of soft poroelastic biosensor for simultaneous epicardial recording and imaging
Source: Nat Commun. 2021 Jun 17;12:3710. doi: 10.1038/s41467-021-23959-3 (PMC8211747; doi:10.1038/s41467-021-23959-3)
Supplement: Supplementary file 3 — Description of Additional Supplementary Files [file 41467_2021_23959_MOESM3_ESM.docx]

**Description of Additional Supplementary Files**

**Supplementary Movie 1.** Spatiotemporal electrophysiological mapping of the ECG signals obtained from the enucleated porcine heart.

**Supplementary Movie 2.** Real-time video of the device placed on the epicardial surface of a murine (left panel) and porcine (right panel) heart.

**Supplementary Movie 3.** In vivo ultrasound video of the device placed on the epicardial surface of a murine heart.

**Supplementary Movie 4.** Real-time ultrasound videos during the removal of the relatively thick (200 µm-thick; top panel) and thin (50 µm-thick; bottom panel) devices from the epicardial surface of a fixed murine heart.

**Supplementary Movie 5.** Real-time display of the postprocessed 3D images reconstructed from the spatiotemporally recorded ECG and ultrasound signals for 0.3 sec after the ligation.

**Supplementary Movie 6.** Real-time ultrasound videos of a murine heart on days 1, 7, and 14 post-implants of the device.
